# Cytogenomics of *Deschampsia* P. Beauv. (Poaceae) Species Based on Sequence Analyses and FISH Mapping of CON/COM Satellite DNA Families

**DOI:** 10.3390/plants10061105

**Published:** 2021-05-30

**Authors:** Alexandra V. Amosova, Lilit Ghukasyan, Olga Yu. Yurkevich, Nadezhda L. Bolsheva, Tatiana E. Samatadze, Svyatoslav A. Zoshchuk, Olga V. Muravenko

**Affiliations:** Engelhardt Institute of Molecular Biology, Russian Academy of Sciences, 32 Vavilov St, 119991 Moscow, Russia; lilitghukasyan90@gmail.com (L.G.); olikys@gmail.com (O.Y.Y.); nlbolsheva@mail.ru (N.L.B.); tsamatadze@gmail.com (T.E.S.); slavazo@mail.ru (S.A.Z.); olgmur1@yandex.ru (O.V.M.)

**Keywords:** *Deschampsia* P. Beauv., satellite DNA, CON/COM sequences, Multiple Sequence Alignment analysis, fluorescence in situ hybridization, chromosomes, interspecific variability

## Abstract

The genus *Deschampsia* P. Beauv. (Poaceae) involves a group of widespread polymorphic species, and many of them are highly tolerant to stressful environmental conditions. Genome diversity and chromosomal phylogeny within the genus are still insufficiently studied. Satellite DNAs, including CON/COM families, are the main components of the plant repeatome, which contribute to chromosome organization. For the first time, using PCR-based (Polymerase Chain Reaction) techniques and sequential BLAST (Basic Local Alignment Search Tool) and MSA (Multiple Sequence Alignment) analyses, we identified and classified CON/COM repeats in genomes of eleven *Deschampsia* accessions and three accessions from related genera. High homology of CON/COM sequences were revealed in the studied species though differences in single-nucleotide alteration profiles detected in homologous CON/COM regions indicated that they tended to diverge independently. The performed chromosome mapping of 45S rDNA, 5S rDNA, and CON/COM repeats in six *Deschampsia* species demonstrated interspecific variability in localization of these cytogenetic markers and facilitated the identification of different chromosomal rearrangements. Based on the obtained data, the studied *Deschampsia* species were distinguished into karyological groups, and MSA-based schematic trees were built, which could clarify the relationships within the genus. Our findings can be useful for further genetic and phylogenetic studies.

## 1. Introduction

The Aveneae/Poeae tribe complex comprises a large number of valuable crops and forage plants [1,2]. Many species of this tribe are polymorphic with a wide geographical distribution, high morphological diversity, and complicated taxonomy [3,4]. The genus *Deschampsia* P. Beauv. (*Poaceae*) comprises more than 30 widespread species, and most *Deschampsia* taxa are highly tolerant to stressful and variable environmental conditions including extreme Arctic and Antarctic regions [5,6,7]. *Deschampsia antarctica* E. Desv. is a perennial grass which occupies a vast area of the Southern Hemisphere including northern Patagonia, sub-Antarctic Islands, the west coast of the Antarctic Peninsula, and the Maritime Antarctic. Moreover, this species is one of two native angiosperms adapted to the severest Antarctic environments [7,8,9,10]. Polar and subpolar ecotypes of *Deschampsia* species could serve as a useful source of genes associated with stress tolerance and environmental adaptation, and investigation of the evolutionary changes which occurred in their genomes could provide insights into the genome history of closely related taxa important for crop breeding [11,12,13,14].

However, genome diversity and comparative chromosomal phylogeny within the genus *Deschampsia* are still insufficiently studied [13,15,16]. Besides, the complicated breeding system peculiar to the evolution process of major taxa within the Aveneae/Poeae tribe (e.g., interspecific and intergeneric hybridization events), could lead to the formation of new allopolyploids [17], and the details of the phylogeny among many genera related to *Deschampsia* and also suprageneric taxa still remain controversial [18]. Moreover, environmental stress factors can induce genetic changes followed by structural chromosomal variations (chromosome rearrangements, mixoploidy, and aneuploidy) in plant species [16,19,20], and therefore, molecular cytogenetic characterization is a key component of the investigation of plant genomes [15,18].

Repetitive DNA (mostly transposable elements and tandem repetitive DNA (satellite DNA)) is one of the main components of genomes of vascular plants [21,22]. The identification and characterization of different satellite DNA families (satDNAs) in genomes of different taxa within the Aveneae/Poeae tribe are currently being studied intensively based on both molecular and cytogenetic levels, and physical mapping of specific satDNAs on individual chromosomes provides information on changes in genome structures which occurred during evolution [22,23,24,25].

CON1, CON2, COM1, and COM2 satDNAs, isolated from the genus *Helictotrichon* [26,27], are shared between many groups of the Aveneae/Poeae tribe complex highlighting their evolutionary significance. Particularly, CON/COM satDNAs were detected in genomes of *Deschampsia cespitosa* (L.) P. Beauv. and *D. antarctica*, and also several species from the genera related to *Deschampsia* [15,24,26,27,28]. Recently, we have performed a comparative molecular cytogenetic characterization of several *Deschampsia* species, which were sampled in different regions including extreme Antarctic (*D. antarctica*) and sub-Arctic areas (*D. sukatschewii* (Popl.) Roshev). The applied new approach, based on multicolor fluorescence in situ hybridization (MC-FISH) with 45S and 5S rDNA and also sequential rapid genomic in situ hybridization (rapid GISH) with genomic DNAs of closely related (*D. antarctica* and *D. cespitosa*) and the most distant (*D. flexuosa* (L.) Trin. (= *Avenella flexuosa* (L.) Drejer)) species helped us clarify the intra- and interspecific variations in their karyotypes and also detect different chromosomal rearrangements [16,19].

The present work aims to characterize CON/COM satDNA families from genomes of eleven *Deschampsia* accessions covering seven species and three plant accessions representing other related genera, and also perform FISH mapping of 45S rDNA, 5S rDNA, and CON/COM satDNAs in karyotypes of *Deschampsia* species to examine the interspecific variability in chromosomal localization of these DNA repeats and specify chromosomal structural changes which occurred within the genus *Deschampsia* during speciation.

## 2. Results

### 2.1. PCR and BLAST Analysis

**Figure 1:**
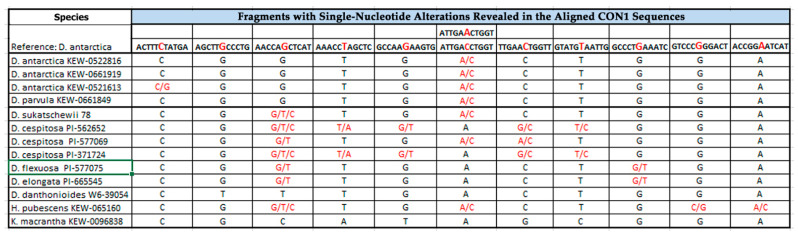
Single-nucleotide alterations (red) revealed in the homologous regions of the obtained CON1 sequences.

**Figure 2:**
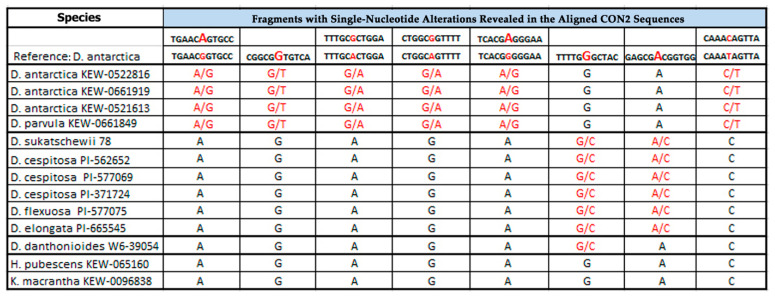
Single-nucleotide alterations (red) revealed in the homologous regions of the obtained CON2 sequences.

**Figure 3:**
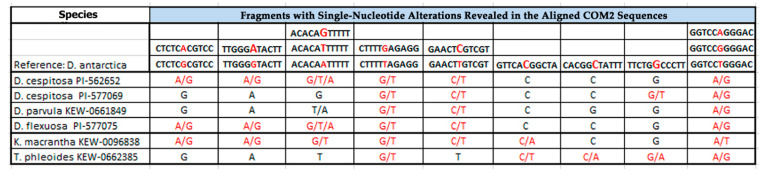
Single-nucleotide alterations (red) revealed in the homologous regions of the obtained COM2 sequences.

For the first time, CON1, CON2, and COM2 satDNAs were identified and classified in genomes of the studied accessions. With the use of a PCR-based technique, CON1 and CON2 sequences were obtained for DNA samples of 13 studied accessions (Figure 1 and Figure 2). COM2 sequences were clearly obtained for six DNA samples (*D. cespitosa* PI-577069, *D. cespitosa* PI-562652, *Deschampsia parvula* (Hook.f.) E. Desv., *D. flexuosa*, *Kolleria macrantha* (Ledeb.) Schult. (syn. *Kolleria cristata* (L.) Pers.), and *T. phleoides*) (Figure 3).

The performed BLAST (Basic Local Alignment Search Tool) analysis revealed high sequence homology between the obtained CON/COM DNAs and the reference DNA sequences of *D. antarctica* reported earlier [24]. CON1 sequences of the studied *Deschampsia* accessions together with *Helictotrichon pubescens* (Huds.) Pilg. (syn. *Avenula pubescens* (Hud.) Dumort.) showed 99% homology with the reference DNA. CON1 sequences of *K. macrantha* and *T. phleoides* exhibited 95% and 93% similarity with the reference sequence, respectively. The homology of CON2 sequences for all available samples was 98–99%. COM2 sequences of the studied *Deschampsia* species exhibited 99% sequence homology with the reference DNA, while COM2 sequences of *K. macrantha* and *T. phleoides* had 93% and 95% similarity with the reference sequence, respectively.

The MSA (Multiple Sequence Alignment) analysis performed within the homologous regions, revealed eleven (in CON1), eight (in CON2), and nine (in COM2) fragments with single-nucleotide alterations (in homozygous and heterozygous states) (Figure 1, Figure 2 and Figure 3). According to the patterns of MSA analyses for CON1 sequences, *D. antarctica* KEW-0522816, *D. antarctica* KEW-0661919, and *D. parvula* demonstrated uniform combinations of alterations (alteration profile) while *D. antarctica* KEW-0521613 had one distinct alteration (Figure 1). CON1 sequences of the other studied samples demonstrated unique profiles. It is noteworthy that CON1 sequences of DNA samples of *D. sukatschewii*, *D. cespitosa* PI-371724, *D. cespitosa* PI-562652, and *H. pubescens* exhibited triplets in heterozygous states (Figure 1).

Based on MSA analyses of CON2 sequences, we determined four groups of DNA samples. The first group included samples of *D. antarctica* KEW-0522816, *D. antarctica* KEW-0661919, *D. antarctica* KEW-0521613, and *D. parvula*. In these samples, the alteration profiles were quite similar to the one observed in the reference CON2 sequence, and differed by just one alteration (column #2) which appeared in the heterozygous state in DNA samples of the first group (in the reference sequence, it was in the homozygous state) (Figure 2).

The second group included DNA samples of *D. sukatschewii*, *D. cespitosa* PI-562652, *D. cespitosa* PI-577069, *D. cespitosa* PI-371724, *D. elongata* (Hook.) Munro, and *D. flexuosa*. The alteration profiles in these samples had only one match (column #2) with the profile of the reference sequence, which was in the homozygous state. Five alterations (columns #1, 3, 4, 5, 8) were heterozygous in the reference sequence, while they were homozygous in samples of this group. The other two variations (columns #6, 7) were heterozygous in samples of the second group but homozygous in the reference CON2 sequence (Figure 2).

The third group included DNA samples of *H. pubescens* and *K. macrantha*. Their profiles exhibited only homozygous alterations and consequently, they matched with the reference CON2 sequence in three (columns #2, 6, 7) homozygous positions and differed in the rest of the alterations (columns #1, 3, 4, 5, 8), which were heterozygous in the reference sequence (Figure 2).

The fourth group included only a DNA sample of *Deschampsia danthonioides* (Trin.) Munro. Its alteration profile matched with the reference CON2 sequence in two homozygous positions (columns #2, 7) and differed in six alterations. Five of them (columns #1, 3, 4, 5, 8) were homozygous in *D*. *danthonoiodes* but they were heterozygous in the reference sequence; the sixth alteration (column #6) was heterozygous in *D*. *danthonoiodes* but it was in a homozygous state in the reference CON2 sequence (Figure 2).

MSA analyses of the COM2 sequences demonstrated that all studied samples had unique alteration profiles (detailed in Figure 3).

**Figure 4:**
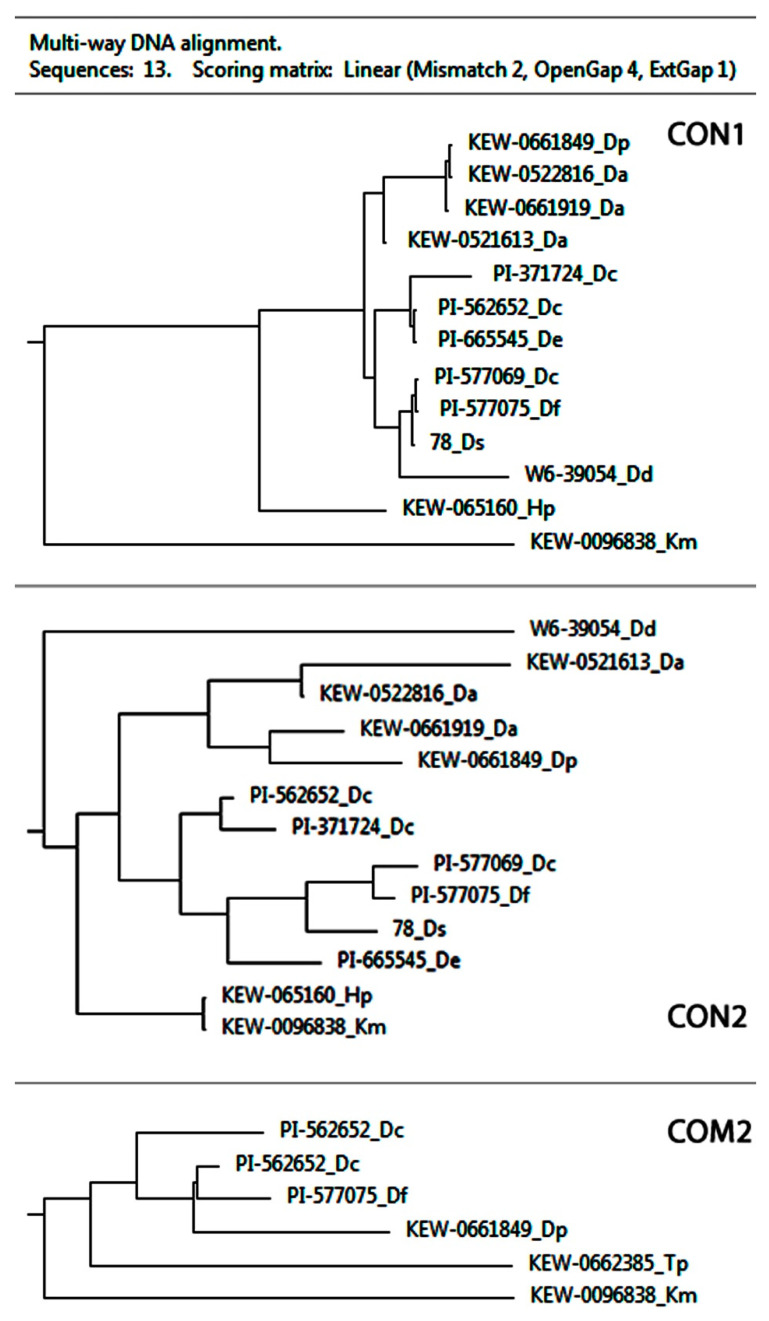
Schematic trees constructed according to the MSA analysis of the aligned CON1, CON2, and COM2 sequences. Da, Dc, Dd, De, Df, Dp, Ds, Hp, Km, and Tp—*D. antarctica*, *D. cespitosa*, *D*. *danthonoiodes*, *D. elongata*, *D. flexuosa*, *D. parvula*, *D. sukatschewii*, *H. pubescens*, *K. macrantha*, and *T. phleoides*, correspondingly.

Based on similarities and differences in the single-nucleotide alterations detected by MSA analyses in the homologous CON1, CON2, and COM2 regions, schematic trees demonstrating the inferred relationships among the studied accessions were built (Figure 4).

### 2.2. Chromosomal Structural Variations in the Studied Accessions

**Figure 5:**
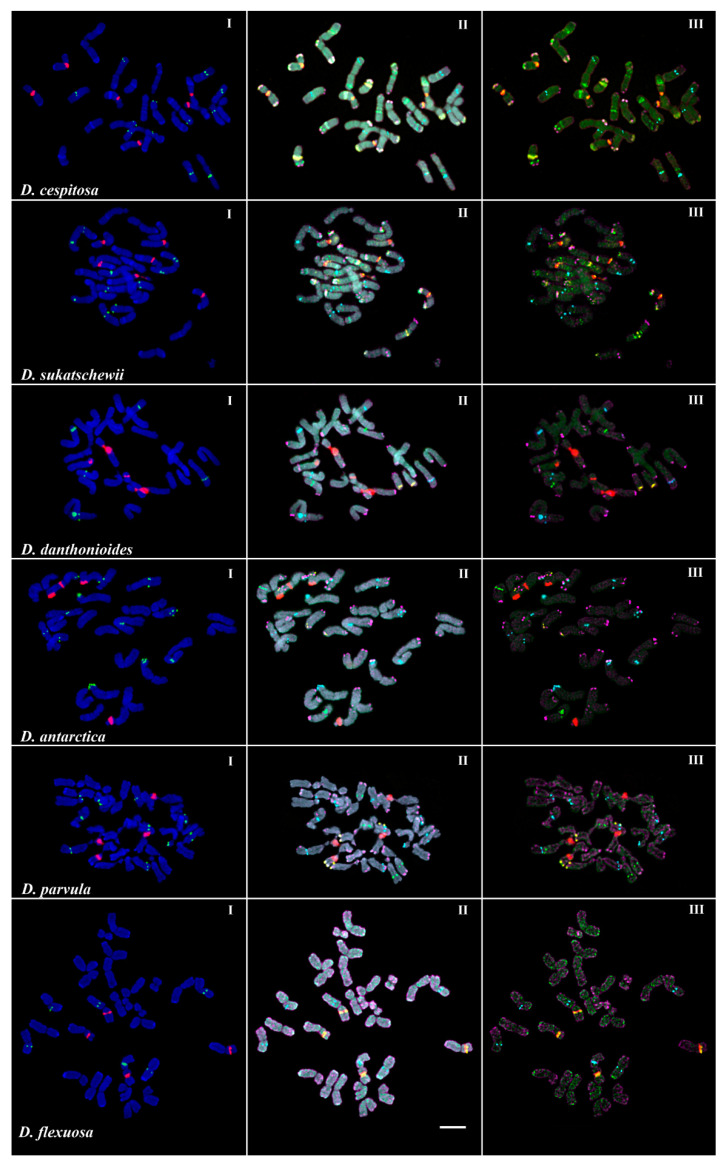
Chromosome spreads of the studied *Deschampsia* species. Merged fluorescent images after MC-FISH with (**I**) 45S rDNA (red) and 5S rDNA (green); (**II**) 45S rDNA (red), 5S rDNA (cyan), CON1 (yellow), CON2 (green), and COM2 (purple) satDNAs and also chromosomal DAPI-staining (gray); (**III**) 45S rDNA (red), 5S rDNA (cyan), CON1 (yellow), CON2 (green), and COM2 (purple) satDNAs. Scale bar—5 μm.

**Figure 6:**
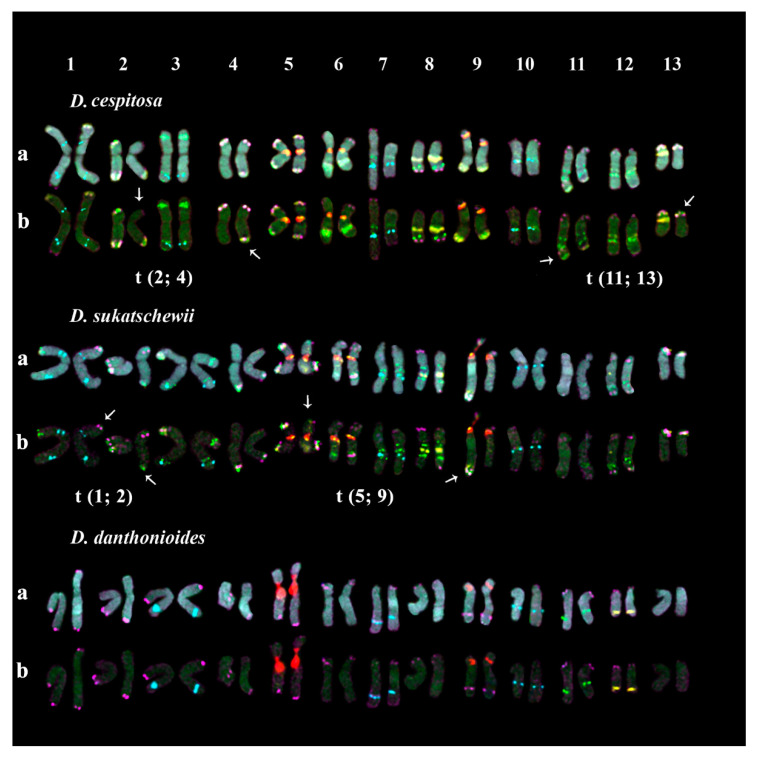
Karyotypes of *D. cespitosa*, *D. sukatschewii*, and *D. danthonioides.* Karyograms of the metaphase plates shown in Figure 5 after MC-FISH with (**a**) 45S rDNA (red), 5S rDNA (cyan), CON1 (yellow), CON2 (green), and COM2 (purple) satDNAs and also chromosomal DAPI-staining (gray) and (**b**) 45S rDNA (red), 5S rDNA (cyan), CON1 (yellow), CON2 (green), and COM2 (purple) satDNAs. Arrows point to chromosome rearrangements.

**Figure 7:**
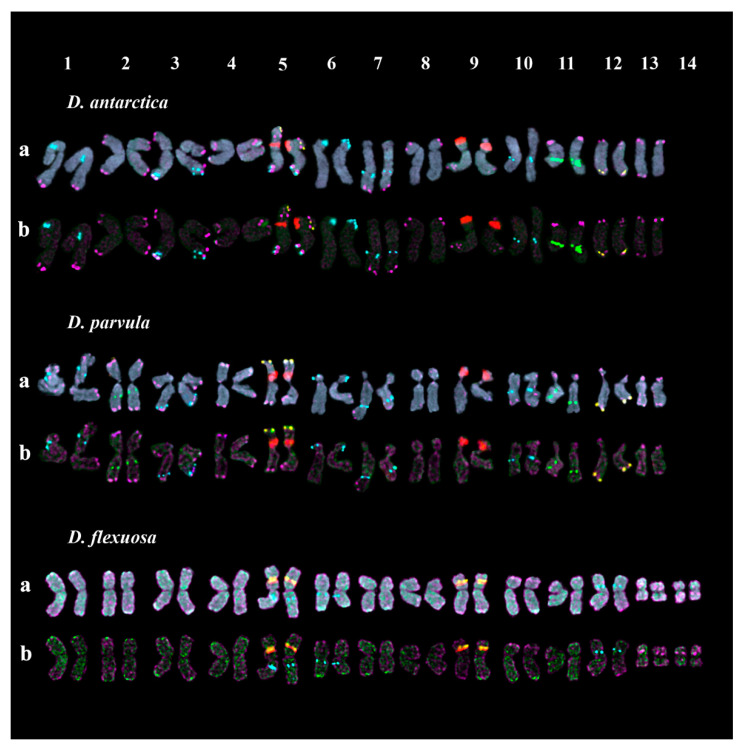
Karyotypes of *D. antarctica*, *D. parvula* and *D. flexuosa.* Karyograms of the metaphase plates shown in Figure 5 after MC-FISH with (**a**) 45S rDNA (red), 5S rDNA (cyan), CON1 (yellow), CON2 (green), and COM2 (purple) satDNAs and also chromosomal DAPI-staining (gray) and (**b**) 45S rDNA (red), 5S rDNA (cyan), CON1 (yellow), CON2 (green), and COM2 (purple) satDNAs.

Karyotype analyses were performed in six species of *Deschampsia: D. antarctica* (KEW-0521613), *D. cespitosa* (PI-371724), *D. danthonioides*, *D. flexuosa*, *D. parvula,* and *D. sukatschewii*. The studied accessions presented diploid karyotype with 2*n* = 26 chromosomes with the exception of *D*. *flexuosa* with 2*n* = 28 chromosomes (Figure 5, Figure 6 and Figure 7).

In karyotypes of the studied *D*. *cespitosa* accession, FISH analysis revealed large 45S rDNA clusters in the secondary constriction regions (short arms) of three satellite (SAT) chromosome pairs 5, 6, and 9. The satellites observed in chromosome pairs 5 and 6 were noticeably longer compared to chromosome pair 9. Additionally, ten 5S rDNA loci were detected on four chromosome pairs: 1 (two loci, in the proximal regions of the short and long arms), 3 (in the distal region of the long arm), and 7 and 10 (in the proximal regions of the long arms) (Figure 6). According to FISH, three satDNA families (CON1, CON2, and COM2) had multiple hybridization signals on chromosomes of the studied *D*. *cespitosa* accession. Both clustered and dispersed localization of CON1 and CON2 satDNAs were observed in different chromosome regions while clusters of COM2 satDNA were localized in the distal regions of most chromosomes. In the secondary constriction regions of the SAT chromosomes, CON1 clusters were co-localized with signals of 45S rDNA. Based on the analysis of chromosome localization of these molecular cytogenetic markers, two chromosomal rearrangements t(2; 4) and t(11; 13) were detected. In both translocations, the subterminal chromosome regions with CON/COM clusters were involved (Figure 6).

In the studied karyotypes of the *D*. *sukatschewii* accession, the pattern of distribution of 45S and 5S rDNA loci was similar to that observed in *D*. *cespitosa*. Multiple clustered and dispersed hybridization signals of CON1 and CON2 satDNAs were observed along the chromosomes though their positions differed slightly from those revealed in karyotypes of *D*. *cespitosa.* In the secondary constriction regions of the SAT chromosomes, CON1 clusters were co-localized with 45S rDNA signals. Clusters of COM2 satDNA were revealed in the distal regions of most chromosomes. In the studied karyotypes of *D*. *sukatschewii*, two chromosomal translocations (t(1; 2) and t(5; 9)) involving the subterminal chromosome regions with CON/COM clusters were detected (Figure 6).

In the studied karyotypes of the *D*. *danthonioides* accession, large 45S rDNA clusters were revealed in the secondary constriction regions (short arms) of two chromosome pairs, 5 and 9 (Figure 6). Clusters of 5S rDNA were detected on chromosome 3 (in the distal region of the long arm), and 7 and 10 (in the proximal regions of the long arms) (Figure 6). In the studied karyotypes, mostly clustered signals of CON/COM satDNAs were observed. Clusters of CON1 and CON2 satDNAs were localized in the long arms of chromosome pairs 12 (in the distal region) and 11 (in the proximal region), respectively. Clusters of COM2 satDNA were observed in the distal regions of most chromosomes (Figure 6).

In karyotypes of the studied *D*. *antarctica* and *D. parvula* accessions, patterns of chromosomal distributions of hybridization sites of 45S rDNA, 5S rDNA, CON1, CON2, and COM2 repeats were rather similar. Large 45S rDNA clusters were located in the secondary constriction regions (short arms) of two chromosome pairs, 5 and 9. The satellite observed in chromosome pair 5 was noticeably longer compared to chromosome pair 9. Clusters of 5S rDNA were revealed in five chromosome pairs: 1 (in the proximal region of the short arm), 3 (in the distal region of the long arm), 6 (in the distal region of the short arm), and 7 and 10 (in the proximal regions of the long arms) (Figure 7). In *D*. *antarctica* mostly clustered signals of CON/COM satDNAs were revealed. In *D. parvula,* clustered and also dispersed signals of CON/COM satDNAs were observed. In both *D*. *antarctica* and *D. parvula,* two CON1 clusters, localized on chromosome pairs 5 (in the distal region of the satellite) and 12 (in the distal region of the long arm), were revealed, and one CON2 cluster was detected in the long arm of chromosome pair 11 (in the proximal region). Besides, in *D. parvula,* the second CON2 cluster was revealed in the long arm of chromosome pair 2 (in the proximal region). In both species, COM2 clusters were revealed in the distal regions of most chromosome pairs (Figure 7).

In the studied karyotypes of the *D*. *flexuosa* accession, large 45S rDNA clusters were revealed in the secondary constriction regions (short arms) of two chromosome pairs, 5 and 9. Three hybridization sites of 5S rDNA were detected on chromosome pair 5 (proximal region, long arm), 6 (proximal region, long arm), and 12 (proximal region, short arm) (Figure 7). Clusters of CON1 were observed on chromosome pairs 5 and 9 in the secondary constriction regions of the SAT chromosomes. Hybridization signals of CON2 and COM2 were mainly dispersed along the different chromosomes with the exception of several COM2 clusters revealed in the short arms (proximal regions) of chromosome pairs 13 and 14 (Figure 7).

## 3. Discussion

In genomes of most plants, satDNAs represent the most abundant fraction of repetitive sequences, and different classes of repetitive DNAs can spread along chromosomes and occupy chromosome-specific regions [21,29]. The evolution rate of repetitive DNAs is considered to be rather high [27,30,31]. The DNA repeats are often involved in chromosome rearrangements which can lead to genomic reorganization and contribute to the genetic diversity during evolution [21,31]. The comparative analysis of patterns of satDNA localization in karyotypes of the related plant species could provide insights into the evolutionary dynamics of chromosome structure variability during speciation within the genus [32]. In the present study, a detailed molecular cytogenetic analysis of *Deschampsia* species, based on MC-FISH with the use of different satDNA families as probes (45S rDNA, 5S rDNA, CON1, CON2, and COM2), allowed us to study the specific patterns of their distribution in karyotypes, identify all homologous pairs of chromosomes and also detect chromosomal rearrangements.

High-copy-number 45S and 5S rDNAs have been found in genomes of all eukaryotes. These rDNA units comprise evolutionarily conserved sequences coding ribosomal rRNAs linked with variable intergenic spacer regions; that is why 45S and 5S rDNAs are widely used in plant phylogenetic studies in different taxonomic groups [33,34,35]. CON1, CON2, COM1, and COM2 satDNA families were initially obtained with restriction enzymes in *Helictotrichon*: CON1 (365 bp) and CON2 (562 bp) in *H. convolutum*, and COM1 (346 bp) and COM2 (476 bp) in *H. compressum* [26,27]. Additionally, CON/COM satDNAs were identified in genomes of other taxa of Aveneae/Poeae tribe complex including genera *Arrhenatherum*, *Helictotrichon*, *Pseudarrhenatherum*, and *Trisetum* [15,28]. The occurrence of CON1, CON2, COM1, and COM2 satDNAs in the genome of *D. cespitosa* were also detected earlier by a dot blot hybridization method [26,27]. However, only CON1, CON2, and COM2 were found in NGS genome sequence data of *D. antarctica* using bioinformatics methods [24]. Our findings are consistent with these data since we also could not reveal the COM1 element in genomes of the studied *Deschampsia* accessions using our approach. Previously, structural heterogeneity related to internal dynamics was detected in the COM1 sequence [29]; therefore, it cannot be excluded that during the evolution of the tribe, COM1 satDNA could change its structure significantly compared to the COM1 element originally detected in *Helictotrichon* [26,27,36]. Alternatively, the COM1 sequence may persist in the *D. antarctica* genome (as well as in genomes of other *Deschampsia* species) but not as a highly repetitive element [24].

In the present study, the obtained CON1, CON2, and COM2 DNA sequences of the studied *Deschampsia* accessions demonstrated high degree of homology with the reference DNA sequences. Our findings are also consistent with the earlier described data on high homology of CON/COM DNA sequences between *D. antarctica* and *D. cespitosa* accessions [25]. At the same time, we revealed different alteration profiles in homologous CON1, CON2, and COM2 regions of the studied samples. Moreover, based on the analysis of CON2 sequences, we observed clear division into groups among the studied *Deschampsia* accessions. These findings can be related to the interspecific genetic divergence which occurred in these satDNAs during the speciation process.

Recently, in *D. antarctica* accessions from various Antarctic and Patagonian populations, intraspecific variability in number and chromosomal localization of FISH signals of CON1, CON2, and COM2 repeats has been observed; this could be related to some evolutionary processes of differentiation in *Deschampsia* species complex in those regions [24]. One of those previously reported polymorphic variants of CON/COM chromosomal localization was basically consistent with our FISH results observed in karyotypes of *D. antarctica* accessions from the Falkland Islands. However, we did not detect intraspecific variability in patterns of chromosomal distribution of CON/COM repeats among the three examined *D. antarctica* accessions. At the same time, patterns of chromosomal distribution of signals of CON1 and CON2 satDNAs observed in the studied *D. cespitosa* accessions differed from the results reported earlier [25,28]. This could be related to high intraspecific genetic diversity revealed in this species [37,38].

In the studied *Deschampsia* species, patterns of chromosome distribution of CON1, CON2, and COM1 signals were different, which was probably related to the processes of speciation within the genus. In three (*D. cespitosa*, *D. sukatschewii*, and *D. flexuosa*) of the six studied species, CON1 clusters were co-localized with 45S rDNA loci in the SAT chromosomes. In the studied accessions of *D. cespitosa* and *D. sukatschewii*, CON2 clusters were associated with 5S rDNA loci. The relationship between chromosome localization of CON2 and 5S rDNA clusters was reported earlier for several taxa of the genera *Helictotrichon* and *Koeleria* (related to *Deschampsia*); however, the low sequence homology between these monomers suggested their independent origin [28].

In the present study, the obtained CON/COM repeats demonstrated clustered and/or dispersed localization along chromosomes, which indicated that they could change their structure and organization in genomes during the species divergence. Particularly, in karyotypes of the studied accessions of *D. cespitosa* and *D. sukatschewii*, CON1 and CON2 signals were abundant demonstrating both clustered and dispersed localization on chromosomes. At the same time, only few CON1 and CON2 clusters were revealed in karyotypes of *D. antarctica*, *D. danthonioides*, and *D. parvula*. In *D. flexuosa*, CON2 signals were dispersed along different chromosomes, and COM2 loci presented both dispersed and clustered localization. Interestingly, in all studied species, COM2 clusters were abundant in number and mainly revealed in subterminal parts of most chromosomes. Subtelomeres are considered to be one of the most dynamic and rapidly evolving regions in eukaryotic genomes [31,39,40]. In different taxa of Aveneae/Poeae tribe complex, these chromosome regions comprise various families of repetitive DNA [41,42], and a significant part (about 60%) of them are AT-rich repeated sequences [30,42,43,44]. It should be noted that CON/COM clusters have AT-richness of 51–56% and often occur in AT-rich chromosome regions [24,45]. The AT-rich terminal satDNAs are suggested to perform some structural functions in a plant genome and contribute to the transition between telomeric domains and internal chromosomal regions [46,47]. Our results are consistent with this suggestion since the chromosomal translocations detected in karyotypes of the studied accessions of *D. cespitosa* and *D. sukatschewii*, involved the subterminal chromosome regions with CON/COM cluster co-localization.

The similarity in monomer sizes and sequences revealed earlier between *D. antarctica* and *Helictotrichon* species (*H. convolutum* and *H. compressum*) indicated that CON1 and CON2 were more conserved than COM2 [24]. The COM2 element in *D. antarctica* (355 bp) was shown to comprise three sub-repeats of approximately 118 bp while COM2 in *H. compressum* (474 bp) consisted of four 120 bp sub-repeats indicating that some sub-structural changes might occur in COM2 DNAs during the divergence of *Deschampsia* species [25,27].

It should be noted that patterns of FISH-based localization of CON1, CON2 and COM2 repeats on chromosomes of the studied *Deschampsia* species were basically similar to the patterns of the satDNA distribution revealed by rapid GISH with genomic DNA of *D. cespitosa* reported previously [16]. These results demonstrate that a rapid GISH approach is a useful tool for comparative karyotype analyses between related diploid plant species. At the same time, the rapid GISH method provided more signals of hybridization indicating the presence of a great number of common DNA repeats in their genomes.

The comparative analysis of chromosome morphology and patterns of distribution of the examined markers allowed us to divide the studied species into four karyological groups: (1) *D. cespitosa*, and *D. sukatschewii*; (2) *D. antarctica* and *D. parvula*; (3) *D. danthonioides*; (4) *D. flexuosa*, which is generally consistent with our previous data on the relationships between these species based on the rapid GISH approach [16].

In the present study, BLAST analysis confirmed the high degree of homology of the obtained CON1, CON2, and COM2 satDNAs with the reference sequences of *D. antarctica*. Based on the MSA analysis of the homologous CON/COM DNA regions, we revealed different groups of *Deschampsia* samples having uniform combinations of single-nucleotide alterations (though the division of the studied species into groups was slightly different). The schematic trees resulted from MSA analyses demonstrated the inferred relationships among the studied accessions based on similarities and differences in the single-nucleotide alterations. Particularly, all accessions of *D. antarctica* and *D. parvula* formed one common group according to CON1 and CON2 sequence alignment. The rest of the species formed different groups depending on the analyzed (CON1 or CON2) sequences.

It is noteworthy that the division of *Deschampsia* species into groups made in accordance with the patterns of CON/COM chromosomal distribution was basically consistent with the schematic trees resulting from the MSA analysis of CON2 sequences. The exception was *D. flexuosa* which belonged to the group of *D. cespitosa* complex according to the MSA analyses of CON1 and CON2 sequences, while it formed a separated group as a result of the analysis of patterns of CON/COM chromosomal localization. Additionally, the relationships between *Deschampsia* species resulting from the patterns of CON/COM chromosomal distribution basically agreed with the taxonomic and molecular phylogenetic studies of the genus *Deschampsia* inferred from nuclear ITS and plastid trnL sequence data reported earlier [18,48]. This highlights the importance of using a complex approach including the analysis of satDNAs and their chromosomal distribution for further taxonomic and phylogenetic studies within *Deschampsia* and other plant genera.

## 4. Materials and Methods

### 4.1. Plant Material

In the present study, we examined eleven *Deschampsia* accessions covering seven species and also three plant accessions representing other genera related to the genus *Deschampsia* (detailed in Table 1). Seeds of the studied species were germinated in Petri dishes with moist filter paper. Then the plants were grown in a greenhouse at 15 °C.

### 4.2. Genomic DNA Extraction, PCR, and Sequencing

Genomic DNAs of the studied samples were isolated from fresh leaves using the Genomic DNA Purification kit (Thermo Fisher Scientific, Waltham, MA, USA). The DNA concentration and purification were assessed with the Implen Nano Photometer N50 (Implen, Munich, Germany).

The CON1, CON2, and COM2 DNA sequences were obtained by PCR amplification with the use of the primer pairs designed earlier for *D. antarctica* [24]. For designing primer pair COM1_F1 (5′-CGGGATAGTACACTTTGGAC-3′) and COM1_R1 (5′-GGAGACCGATGGATTTTC-3′), we used the COM1 DNA sequence of *Helictotrichon compressum* (ID pCOM1_1) (accession number Z68777.1) and Clone Manager 10 software (Sci Ed Software, Westminster, CO, USA).

The fragments of the target satDNAs were amplified under the following conditions: the reaction mix contained 1 μL of template DNA (at least 30 ng), 5 pM of each primer, 1U of Taq DNA polymerase (Sintol, Moscow, Russia), 2.5 μL of 10 × Taq buffer (Sintol, Moscow, Russia), 1.5 μL of 25 mM MgCl_2_ (Sintol, Moscow, Russia), and 0.5 mM of each dNTP (Silex, Moscow, Russia) in a total volume of 25 μL. The reactions were performed with the use of a T100 thermal cycler (Bio-rad, CA, USA) under the following cycle conditions: 3 min at 95 °C, followed by 35 cycles of 20 s at 95 °C, 20 s at 60 °C, and 20 s at 72 °C with 5 min of a final extension at 72 °C.

A total of 5 μL of each amplified PCR products were separated by electrophoresis (2.0% agarose gel containing 0.1% ethidium bromide at 100 V for 25 min) and then visualized with the Bio-Rad Gel Doc XR + UV transilluminator (Bio-Rad, Hercules, CA, USA). The PCR product of COM1 satDNA was not used hereafter due to its unsatisfactory quality.

In case of the presence of one clear band on the gel, the rest of the PCR product (20 μL) was purified by ethanol precipitation for further sequencing. If there was more than one band on the gel, the rest of the PCR product (20 μL) underwent electrophoresis for further separation, then the band of the desired length was cut out from the gel and extracted using the QIAquick Gel Extraction Kit (Qiagen, Hilden, Germany).

The obtained and purified fragments of the CON1, CON2, and COM2 (with lengths of 333, 538, and 322 base pairs, respectively) were then Sanger sequenced using the automatic Applied Biosystems (ABI) 3730 DNA sequencer (Applied Biosystems, Foster City, CA, USA). The obtained sequences were analyzed with the use of Chromas 2.5 software (Technelysium Pty, South Brisbane, Australia). The homology of the obtained sequences with the reference DNAs was estimated by BLAST (Basic Local Alignment Search Tool) (NCBI, MD, USA). As reference DNA sequences, we used the CON1, CON2, and COM2 sequences of *D. antarctica* reported earlier [24]. Further MSA (Multiple Sequence Alignment) analyses were performed for detection single-nucleotide alterations in the aligned sequences of the obtained and reference CON/COM DNAs. Then, based on the MSA analyses performed in the homologous regions of the obtained CON/COM sequences, schematic trees were built with the use of Clone Manager 10 software (Sci Ed Software, Westminster, CO, USA).

### 4.3. Chromosome Spread Preparation

For accumulation of mitotic divisions, root tips (0.5–1 cm long) were incubated in ice water for 24 h and then fixed in the ethanol:glacial acetic acid fixative (3:1) for 48 h at room temperature. Fixed roots were stored in the fixative at −20 °C before use.

Before spread preparation, the roots were put into 1% acetocarmine solution in 45% acetic acid for 40 min. Then, on the object plate, the tip caps with root meristem were cut, macerated in a drop of 45% acetic acid, and covered with a cover slip. Then squashed chromosome preparations were made. The obtained preparations were frozen in liquid nitrogen to remove the cover slips, then dehydrated in the ethanol series (70, 85, and 96%) and stored in 96% ethanol at –20 °C before use.

### 4.4. Fluorescence In Situ Hybridization

- In FISH assays, the following probes were used:
- pTa71 containing a 9 kb long DNA sequence of common wheat enclosing 18S-5.8S-26S (45S) rDNA [49];
- pTa794 containing a 420 bp long DNA sequence of wheat containing 5S rDNA [50];
- CON1, CON2, and COM2 sequences obtained by the PCR-based technique.

These DNA probes were labeled directly with fluorochromes Aqua 431 dUTP, Red 594 dUTP, or Green 496 dUTP (Enzo Life Sciences, Lausen, Switzerland) by Nick translation according to manufacturers’ protocols. The several sequential MC-FISH procedures were conducted with various combinations of these labeled DNA probes as described previously [51,52]. After FISH, chromosome slides were stained with 4′,6-diamidino-2-phenylindole (DAPI) dissolved in the Vectashield mounting medium (Vector Laboratories, Burlingame, CA, USA) to a final concentration of 0.1 μg/mL.

### 4.5. Chromosomal Analysis

The chromosome slides were examined with the use of the Olympus BX61 epifluorescence microscope (Olympus, Tokyo, Japan) coupled with a monochrome CCD camera (Snap, Roper Scientific, Tucson, AZ, USA). Chromosome images were collected in grayscale channels and pseudo-colored. Then they were processed using conventional Adobe Photoshop 10.0 (Adobe Systems, Birmingham, AL, USA) and VideoTesT-FISH 2.1 (IstaVideoTesT, St. Petersburg, Russia) software programs. At least 5 plants and 15 metaphase plates were examined in each sample. Based on the chromosome size and morphology and also distribution of chromosome markers, chromosome pairs in karyotypes were identified using the cytological nomenclature proposed earlier [16].

## 5. Conclusions

The obtained CON1, CON2, and COM2 satDNAs demonstrated high sequence homology between the studied *Deschampsia* species though their chromosomal distribution was different. These satDNAs can be used as specific markers for identification of chromosome pairs and detection of chromosomal rearrangements relevant for investigation of the genomic relationships within the genus *Deschampsia*. Our findings highlight the importance of changes which occurred in satDNA structures and their chromosomal disposition for clarifying the species divergence within this complex genus and also between related taxa. The obtained unique information on cytogenomic structures of *Deschampsia* species can serve as a basis for further genetic and phylogenetic studies, as well as for plant breeding.

## Figures and Tables

**Table 1:** The list of the studied plant accessions.

Species	Catalog Number/Origin	Seed Source
*Deschampsiaantarctica* E. Desv.	KEW-0522816/Falkland Is., St. Georgia	Seed Conservation Department, Royal Botanic Gardens, Kew, UK
*Deschampsiaantarctica* E. Desv.	KEW-0661919/Falkland Is., Weddle	Seed Conservation Department, Royal Botanic Gardens, Kew, UK
*Deschampsiaantarctica* E. Desv.	KEW-0521613/Falkland Is., St. Georgia	Seed Conservation Department, Royal Botanic Gardens, Kew, UK
*Deschampsia cespitosa ssp. beringensis* (Hultén) W.E. Lawr.	PI-562652/Alaska, USA	Western Regional Plant Introduction Station, USDA ARS NPGS, Pullman, WA, USA
*Deschampsia cespitosa* (L.) P. Beauv.	PI-371724/Alaska, Valdez. USA	Western Regional Plant Introduction Station, USDA ARS NPGS, Pullman, WA, USA
*Deschampsia cespitosa* (L.) P. Beauv.	PI-577069/Great Britain	Western Regional Plant Introduction Station, USDA ARS NPGS, Pullman, WA, USA
*Deschampsia danthonioides* (Trin.) Munro	W6-39054/Washington, USA	Western Regional Plant Introduction Station, USDA ARS NPGS, Pullman, WA, USA
*Deschampsia elongata* (Hook.)	Munro PI-665545/Oregon, USA	Western Regional Plant Introduction Station, USDA ARS NPGS, Pullman, WA, USA
*Deschampsia*. *flexuosa* (L.) Trin. (= *Avenella flexuosa* (L.) Drejer)	PI-577075/Wales, UK	Western Regional Plant Introduction Station, USDA ARS NPGS, Pullman, WA, USA
*Deschampsia parvula* (Hook.f.) E. Desv.	KEW-0661849/Falkland Is.	Seed Conservation Department, Royal Botanic Gardens, Kew, UK
*Deschampsia sukatschewii* (Popl.) Roshev	78/Altai Mountains, RF	Laboratory of genetic resources of fodder plants, Federal Williams Research Center of Forage Production and Agroecology (FWRC FPA)
*Helictotrichon pubescens* (Huds.) Pilg. (syn. *Aenula pubescens* (Hud.) Dumort.)	KEW-065160/England, UK	Seed Conservation Department, Royal Botanic Gardens, Kew, UK
*Kolleria macrantha* (Ledeb.) Schult. (syn. *Kolleria cristata* (L.) Pers.)	KEW-0096838/Greece	Seed Conservation Department, Royal Botanic Gardens, Kew, UK
*Tricetum phleoides* (d’Urv.) Kunth.	KEW-0662385/Falkland Is.	Seed Conservation Department, Royal Botanic Gardens, Kew, UK

## Data Availability

All data are contained within the article.
